# Breast reconstruction with single-pedicle TRAM flap in breast cancer patients with low midline abdominal scar

**DOI:** 10.1038/srep29580

**Published:** 2016-07-13

**Authors:** Jun-Dong Wu, Wen-He Huang, Si-Qi Qiu, Li-Fang He, Cui-Ping Guo, Yong-Qu Zhang, Fan Zhang, Guo-Jun Zhang

**Affiliations:** 1 The Breast Center, Cancer Hospital of Shantou University Medical College, 7 Raoping Rd. Shantou 515041, Guangdong, China; 2 Changjiang Scholar’s Laboratory, Cancer Hospital of Shantou University Medical College, Guangdong, China

## Abstract

Breast reconstruction with transverse rectus abdominis myocutaneous (TRAM) flap is challenging in patients with low midline abdominal scar. In this study, we aimed to investigate the clinical feasibility of immediate breast reconstruction using single-pedicle TRAM (SP-TRAM) flaps in patients with low midline abdominal scar. There were 4 strict selection criteria: 1) presence at least 3 perforators on the pedicle side; 2) perforators with regional average flow velocity of >20 cm/s; 3) upper edge of the abdominal scar at least 4 cm from the umbilicus; and 4) scar age >1 year. Eight breast cancer patients with low midline abdominal scar (scar group) and 20 without (control group) underwent immediate breast reconstruction with SP-TRAM flaps consisting of zone I and III and zone II tissues. Flap complications, donor-site complications, and cosmetic results were compared between the two groups. All flaps survived and both groups presented similar flap and donor site complications, including fat necrosis, seroma, hematoma, infection, delayed wound healing, and abdominal hernia, and patients in both groups had similar aesthetic results (p > 0.05). Thus, the study demonstrated that breast reconstruction using SP-TRAM flap was a safe approach in carefully selected patients with low midline abdominal scar.

Transverse rectus abdominis myocutaneous (TRAM) flap and its variations are considered the best available options for autologous breast reconstruction after mastectomy^1^,^2^,^3^,^4^. Abdominal scars from previous surgeries often present a challenge when TRAM flap is desired for breast reconstruction^1^,^2^,^5^,^6^,^7^,^8^. In patients with low midline abdominal scars, for instance, tissue perfusion across the midline scar is unreliable, which may lead to flap necrosis, fat necrosis, or wound breakdown at the donor site^2^,^5^,^7^.

Nonetheless, TRAM flaps can still be appropriate for breast reconstruction even in the presence of a low midline abdominal scar^9^. Various operative strategies have been suggested to improve flap survival and reduce donor-site complications in these patients. A hemi-TRAM flap can be used safely in patients with low midline abdominal scar^5^,^9^,^10^, but it will limit the volume of the reconstructed breast^1^,^2^,^5^,^10^. Thus, other techniques, including double-pedicle or free TRAM flaps and deep inferior epigastric perforator (DIEP) flaps, which can augment tissue perfusion across midline scars, have also been proposed^5^,^10^. However, all of these methods are complex and tedious, and furthermore, the use of complicated microsurgical techniques may not be practical in all clinical situations^2^,^5^.

In this study, we performed breast reconstruction using single-pedicle TRAM (SP-TRAM) flaps in selected patients with low midline abdominal scar. In order to guarantee sufficient flap volume at the donor site, the locations and peak systolic flow velocities of the perforators were determined by preoperative color-flow duplex scanning for each patient. We compared the outcomes of SP-TRAM breast reconstruction in patients with or without low midline abdominal scar in 8 (scar group) and 20 (control group) patients, respectively, and this small series demonstrated the safety and feasibility of SP-TRAM flaps in patients with low midline abdominal scar.

## Results

### Patient characteristics

There were no significant differences in age, body mass index (BMI), smoking history, diabetes mellitus, and neo-adjuvant chemotherapy or preoperative radiotherapy between the scar and control groups, and no significant difference between groups in pathological characteristics (Table 1).

All patients in both groups received SP-TRAM flaps (including zones I, III, and II) showing sufficient blood supply in zone II. During mean follow-up of 24.3 months (range, 8 to 34 months) all patients remained alive, and most remained disease-free, excepting one patient in the control group who developed brain metastasis.

### Cosmetic effects

**Figure 1:**
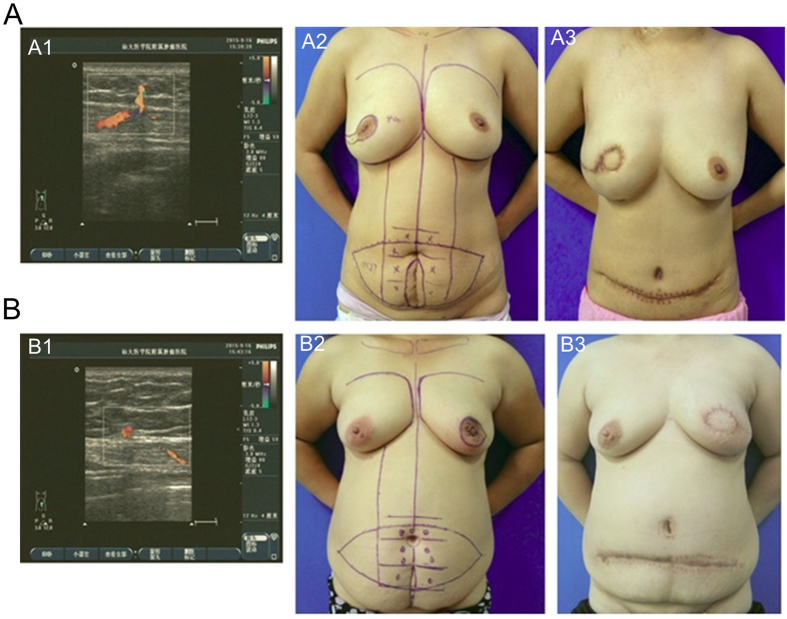
(**A**) A 37-year-old woman with invasive ductal carcinoma in the right outer quadrant close to the areola. A1: Location and peak systolic flow velocities of the perforators were assessed with pre-operative color-flow duplex ultrasound scanning. A2: The patient had a previous low midline abdominal scar and breast reconstruction with a single-pedicle transverse rectus abdominis myocutaneous (SP-TRAM) flap was planned. A3: The patient underwent mastectomy with immediate reconstruction using single-pedicle TRAM flap and was followed for 20 months. (**B**) A 50-year-old woman with invasive ductal carcinoma in the right central quadrant. B1: The perforator locations and peak systolic flow velocities were detected by pre-operative color-flow duplex scanning. B2: The patient had previous low midline abdominal scar and was scheduled for mastectomy with immediate SP-TRAM flap breast reconstruction. B3: The same patient at 6 months after surgery.

The cosmetic effects were recorded as “excellent-good” in 75.0% (6/8) and “fair-poor” in 25.0% (2/8) of patients in the scar group and as “excellent-good” in 85.0% (17/20) and “fair-poor” in 15.0% (3/20) patients in the control group (Fig. 1). The differences between groups were not significant (p = 0.61).

### Flap-related complications

One patient in each group had partial flap loss. There were no instances of total flap loss. One patient (1/8; 12.5%) in the scar group and 3 patients (3/20; 15%) in the control group developed fat necrosis; the rates of partial flap loss and fat necrosis were not statistically different between the two groups (p = 1.00). No patient in either group developed seroma, hematoma, or flap-site infection, and there was no significant difference in the overall complication rates between the two groups (Table 2).

### Donor-site related complications

One patient in each group (1/8, 12.5%; 1/20, 5%) developed incisional infection; the rates of fat necrosis and delayed wound healing in each group were also 1/8 (12.5%) and 1/20 (5%). One patient in the control group (1/20, 5%) developed hematoma. Abdominal hernia or bulge and seroma did not occur in either group. The overall rates of donor-sites complications were not statistically different between the two groups (p = 0.37) (Table 3).

## Discussion

In most cases, abdominal scar does not preclude the use of the TRAM flap, but it may determine how much tissue is available for use in the breast reconstruction^2^, especially in patients with low midline abdominal scar^2^,^7^,^11^. Flap perfusion across the midline scar in these patients is potentially insufficient^1^,^2^,^5^,^12^, but hemi-TRAM flaps may not be sufficient for reconstruction of a large breast, while hemi-TRAM flaps with larger volumes may cause greater tension in the abdominal wound and prevent closure. Therefore, zone II tissues across the midline scar may have to be included when designing a TRAM flap.

Several alternate strategies have been developed for matching the contralateral breast in these cases, including creation of bilateral-pedicled TRAM flaps^2^,^5^,^11^, developing the flap higher in the abdomen, and anastomosing a contralateral deep inferior epigastric artery^2^. While these techniques may augment flap perfusion across the midline scar, they are technically complicated, require long operation times, and may cause hemodynamic crisis. Thus, they are not practical in all patients with low midline abdominal scar. In the present study, which included Chinese breast cancer patients with and without low midline abdominal scar, we used SP-TRAM flaps for immediate breast reconstruction after mastectomy, which simplified the surgical procedure and minimized operative injury. The results demonstrated that it is feasible to use SP-TRAM flaps in in strictly selected patients who have relatively small breasts without increasing the risk of post-operative complications.

Previous abdominal wall surgery is known to alter the vascular anatomy and architecture, specifically of the cutaneous vasculature^13^. According to the delay phenomenon, ischemia due to abdominal wall incision is usually compensated over time by the functional dilation of adjacent vessels^7^,^13^,^14^. In keeping with this phenomenon, Heller *et al*.^2^ found that perfusion across the midline scar to the contralateral side was surprisingly good, and zone II tissues were usable for breast reconstruction. Santamaria *et al*. reported that contrast material was detected in zone II across the midline scar in 7 patients within minutes of contrast injection into the inferior epigastric artery^15^. Han *et al*. investigated the effects of an abdominal midline incision on the survival of TRAM flaps in a rat model and found that the scars experienced changes similar to those observed during the delay phenomenon in humans, with increasing size and density of the subdermal plexus^16^. Thus, the delay phenomenon may also play an important role in the regenerative potential of perforators, obstructed vessels, and the subdermal vascular plexus. Recent studies have indicated that there is a significant increase in microvascular density in the subdermal layer during the repair process following injury^16^,^17^, which was sustained for up to 52 weeks after the injury. It is assumed that the older the scar, the greater the extent of neo-vascularization^16^,^17^, so the age of the scar should be at least 1 year, as in our study, to allow regeneration of transected perforators. Rand *et al*.^18^ have recommended that the donor site should have >3 perforators with flow velocities if >20 cm/s in order to support an SP-TRAM flap. Because separating the umbilicus from the flap will damage the perforators across periumbilical areas, we recommend that distance from the upper edge of the low midline abdominal scar to the umbilicus should be >4 cm. Moreover, we routinely resect zone IV tissues while preserving the zone II tissues.

Color duplex ultrasonography, CT angiography and MR angiography methods are useful for preoperative assessment of flap perfusion in the presence of abdominal scar^19^,^20^. Intraoperative laser-assisted indocyanine green (ICG) angiography is also used to measure the perfusion of the TRAM flap before flap harvest and transfer^21^,^22^. While color duplex ultrasound can be a simple and effective modality for examining the patient’s vascular anatomy and determining the number of perforators^19^. In our study, this modality allowed visualization of perforators >1 mm in diameter, localization of major perforators, and determination of peak systolic flow velocities in all patients.

Rates of flap necrosis after breast reconstruction have ranged from 5.0% to 35.0%^3^,^8^ overall, and from 11.7% to 16.1% for free TRAM flap with previous abdominal scar^6^,^8^,^23^. In our study, strict selection of patients resulted in a rate of flap necrosis in the scar group (25.0%) that was not statistically different from that in the control group (20.0%) (p = 1.00). Anastomotic branches of superior and deep inferior gastric arteries are most abundant in the periumbilical area^18^. In our study, the perforators were most frequently identified in zone II which was within 4 cm below umbilicus (Tables 4 and 5). Our flaps were designed with a superior border 2.0 cm above the umbilicus and an inferior border 10 cm below the umbilicus in order to obtain a larger scar-free and vascularized area^2^ (Fig. 1), and the tendons of rectus abdominis were preserved to avoid injury to the perforators. These measures are key to flap survival and can potentially reduce rates of fat necrosis and flap loss. In our study, fat necrosis occurred in 1 patient (1/8; 12.5%) in the scar group and in 3 patients (3/20; 15.0%) in the control group, and the overall rates of flap-related complications were not significantly different between the two groups.

There are still controversies in terms of donor-site complications. Some have reported increased risk of abdominal complications^6^,^24^,^25^ while others have not^1^,^8^,^11^,^23^,^26^. In our study, there were no differences in the rates of incision infection (12.5% versus 5.0%, p = 0.50) or delayed wound healing (12.5% versus 5.0%, p = 0.50) between the scar group and the control groups, and there was no significant difference in the overall complication rates (37.5% versus 20.0%, p = 0.37), which indicates that SP-TRAM flaps are safe and feasible even in the presence of low midline abdominal scar. Use of a Prolene mesh repair for the abdominal defect has been associated with similar complication rates in patients undergoing SP-TRAM flap and DIEP flap breast reconstruction^27^. In the present study, we used mesh repair for the rectus sheath defects and there were no instances of subsequent abdominal wall hernia or bulge during the follow-up period. We continue to recommend the use of mesh to repair the abdominal wall defect in patients receiving pedicled flaps^28^,^29^.

Cosmetic effects were evaluated independently by three surgeons, excluding the operating surgeons, and were comparable in the scar group and the control group, which is consistent with previous reports^7^,^11^.

In comparison with free TRAM flaps or DIEP flaps, breast reconstruction with SP-TRAM flap allows shorter operation times and does not require microvascular anastomosing techniques. Thus, even with the development of these more complex and purportedly more beneficial techniques, the SP-TRAM flap remains a reliable alternative for breast reconstruction that yields a high degree of patient satisfaction^3^,^30^, and the good clinical results in our patients confirm that breast reconstruction with the SP-TRAM flap is feasible in patients with low midline abdominal scar.

## Conclusions

In this study, 8 patients with low midline abdominal scar who met strict selection criteria underwent successful SP-TRAM flap breast reconstruction. Our clinical findings have demonstrated that in carefully selected patients, tissues across the midline scar will have a relatively sufficient blood supply and can be safely used for SP-TRAM flap breast reconstruction.

### Patients and Methods

A total of 28 consecutive patients who underwent unilateral breast reconstruction with SP-TRAM flap after mastectomy from April 2013 to October 2015 at the Breast Center of the Cancer Hospital of Shantou University Medical College, 8 with previous low midline abdominal scar (scar group) and 20 without low midline abdominal scar (control group), were included in the study. In the scar group, the average length of the vertical low midline abdominal scar was 9.3 cm (range, 5 cm to 14.5 cm). The mean age of the midline abdominal scar was 13.5 years (range, 4 to 28 years). The clinico-pathological characteristics of both groups are shown in Table 1.

Preoperative blood supply was evaluated by color-flow duplex ultrasound imaging using the Philips IU-22 device, and locations and peak systolic flow velocities of major perforator vessels of the rectus abdominis muscles were identified in both groups. In the scar group the abdomen was divided into quadrants centered around the umbilicus so that perforator locations could be placed within a Cartesian grid^18^. As previously reported, four zones were divided with 4 cm height by designating the umbilicus as the zero position^18^. Locations of major perforators along the superior and inferior epigastric arteries were identified, and the peak systolic flow velocities of the perforators were measured in each zone, as shown in Tables 4 and 5. Patients with low-midline abdominal scars met the following selection criteria for breast reconstruction with SP-TRAM flap: 1) at least 3 perforators in the flap territory; 2) average peak systolic flow velocities of the perforators of >20 cm/s; 3) upper edge of the midline scar >4 cm from the umbilicus; and 4) scar age >1 year.

### Surgical Technique

All 28 patients underwent breast reconstruction with SP-TRAM flap performed by the same surgical team. In 8 patients with low-midline abdominal scar, the flaps were outlined on the lower abdomen including the position of the perforators identified by color Doppler. The upper edge of the abdominal incision was 2 cm above the umbilicus, so that the more superior flap was scar-free in order to allow a larger skin bridge. The lower incision included 1/2 to 2/3 of the upper portion of the scar according to the volume of the reconstructed breast and the tension required to permit closure of the abdominal donor site. During the procedure, the skin and subcutaneous tissue of the flap were dissected to the anterior rectus sheath and the upper abdominal subcutaneous tissues were separated in order to form a tunnel to the chest wall, and the umbilicus was separated and retained on the anterior rectus sheath. The anterior wall of the rectus sheath was separated and the tendon was reserved on the muscle. The skin color and bleeding condition of the flap were observed for 15 to 20 min, with particular attention to the tissues of zone II across the midline scar, and perfusion of the flap was assessed by capillary refill. If the perfusion was deemed sufficient, flaps including zone II could be harvested and transferred for breast reconstruction with TRAM flaps, otherwise, the tissues were harvested as hemi-TRAM flaps.

The flaps were rotated through the subcutaneous tunnel to the chest defect, and the defect of the anterior wall of the rectus sheath was repaired with a mesh. The umbilicus was pulled out to the abdominal wall through a small incision and sutured, and, following drain placement, the abdominal wall closed. Finally, the flap was shaped to match the contralateral breast.

### Post-operative Evaluation and Follow-up

Immediate post-reconstructive complications were recorded as flap or donor-site related. Flap-related complications were total flap loss, defined as complete necrosis of the skin and fat; partial flap loss, defined as ischemic tissue loss exceeding 25% or fat necrosis characterized by subcutaneous firmness >7.5 cm in diameter^6^,^30^; fat necrosis, defined as loss of a portion of the adipose component with subcutaneous firmness of at least 2 cm to 7.5 cm in diameter^1^,^6^,^30^,^31^; seroma formation, defined as palpable fluctuation of subcutaneous tissues requiring suction or drainage; and wound dehiscence and wound infection, defined as redness, swelling, and exudate and requiring antibiotics^1^,^23^. Donor-site complications included seroma, wound infection, fat necrosis, postoperative hematoma requiring evacuation, delayed wound healing, and abdominal wall bulge or hernia requiring operative repair^24^,^30^.

All patients had follow-up examinations every 3 months, and cosmetic effects were evaluated by using postoperative photographs obtained at 6 months after surgery. The cosmetic effects were assessed by a team of expert surgeons that excluded the operating surgeons. Four items (symmetry, volume, position of the infra-mammary fold, and ptosis) were assessed, and cosmesis was defined as excellent, good, fair, or poor according to the Lowery Scaling System^32^. To minimize bias in our results, the cosmetic effects were rated as either “excellent-good” or “fair-poor.”

### Statistical Analysis

SPSS version 13.0 software (SPSS, Inc., Chicago, IL, USA) was used for statistical analysis, with comparison of discrete variables by Pearson chi-square or Fisher’s exact test, and comparison of means by t-test. A value of p < 0.05 was considered significant.

### Ethical approval

This study was approved by the Ethics Committee of the Cancer Hospital of Shantou University Medical College and was performed in accordance with the ethical standards of the 1964 Declaration of Helsinki and all subsequent revisions. All persons mentioned in the paper gave informed consent prior to their inclusion in the study.

## Additional Information

**How to cite this article**: Wu, J.-D. *et al*. Breast reconstruction with single-pedicle TRAM flap in breast cancer patients with low midline abdominal scar. *Sci. Rep.* **6**, 29580; doi: 10.1038/srep29580 (2016).

## Figures and Tables

**Table 1:** Patient characteristics.

Demographics	Scar Group (n = 8)	Control Group (n = 20)	t	χ^2^	*P*
Mean age (yr)	42.3 ± 6.6	38.4 ± 5.3	−1.64		0.11
BMI (kg/m^2^)	24.1 ± 2.4	22.2 ± 2.3	−1.95		0.06
Diabetes	0 (0.0%)	1 (5.0%)		–	1.00
Smokers	1 (12.5%)	0 (0.0%)		–	0.29
NAC	1 (12.5%)	1 (5.0%)		–	0.50
PR	0 (0.0%)	0 (0.0%)			–
Histology				–	0.79
DCIS	0 (0.0%)	2 (10.0%)			
LCIS	0 (0.0%)	1 (5.0%)			
IDC	7 (87.5%)	15 (75.0%)			
ILC	1 (12.5%)	0 (0.0%)			
mucinous	0 (0.0%)	1 (5.0%)			
Medullary	0 (0.0%)	1 (5.0%)			
Histological grade				–	0.27
1	2 (25.0%)	1 (5.0%)			
2	4 (50.0%)	7 (35.0%)			
3	2 (25.0%)	9 (45.0%)			
*In situ* carcinoma	0 (0.0%)	3 (15.0%)			
pTNM stage				–	0.38
0	0 (0.0%)	3 (15.0%)			
I	2 (25.0%)	3 (15.0%)			
II	5 (62.5%)	14 (70.0%)			
III	1 (12.5%)	0 (0.0%)			
Molecular subtype				–	0.76
Luminal A	2 (25.0%)	6 (30.0%)			
Luminal B1	4 (50.0%)	5 (25.0%)			
Luminal B2	0 (0.0%)	3 (15.0%)			
HER-2 overexpression	1 (12.5%)	2 (10.0%)			
Triple negative	1 (12.5%)	4 (20.0%)			

Note: Fisher’s exact tests.

Abbreviations: BMI, body mass index; NAC, neoadjuvant chemotherapy; PR, preoperative radiotherapy; DCIS, Ductal carcinoma *in situ*; LCIS, Lobular carcinoma *in situ*; IDC, Invasive ductal carcinoma; ILC, Invasive lobular carcinoma.

**Table 2:** Flap-related complications.

	Scar group (n = 8)	Control group (n = 20)	χ^2^	*P*
Overall	2 (25.0%)	4 (20.0%)		1.00
Completely flap loss	0 (0.0%)	0 (0.0%)		−
Partial flap loss	1 (12.5%)	1 (5.0%)		0.50
Fat necrosis	1 (12.5%)	3 (15.0%)		1.00
Seroma	0 (0.0%)	0 (0.0%)		–
Hematoma	0 (0.0%)	0 (0.0%)		–
Infection	0 (0.0%)	0 (0.0%)		–

Note: Fisher’s exact tests.

**Table 3:** Donor site complications.

	Scar group (n = 8)	Control group (n = 20)	χ^2^	*P*
Overall	3 (37.5%)	4 (20.0%)		0.37
Seroma	0 (0.0%)	0 (0.0%)		−
Infection	1 (12.5%)	1 (5.0%)		0.50
Fat necrosis	1 (12.5%)	1 (5.0%)		0.50
Hematoma	0 (0.0%)	1 (5.0%)		1.00
Delayed wound healing	1 (12.5%)	1 (5.0%)		0.50
Hernia/bulge	0 (0.0%)	0 (0.0%)		−

Note: Fisher’s exact tests.

**Table 4:** Frequency of perforators by side and zone in 8 patients (scar group) (%).

	Zone I	Zone II	Zone III	Zone IV	Total
Right	10	12	10	7	39
Left	9	13	10	6	38
Total	19	25	20	13	77
Percent	24.7	32.5	26.0	16.9	

**Table 5:** Peak systolic flow velocity in TRAM flap perforators in 8 patients (scar group)(cm/s).

	Zone I		Zone II		Zone III		Zone IV	
	Right	Left	Right	Left	Right	Left	Right	Left
Minimum	6.2	5.8	5.4	5.0	4.5	4.5	3.8	4.2
Maximum	42.5	42.8	52.5	45.4	40.4	38.5	21.3	10.4
Mean Flow	20.9	22.3	25.5	21.8	18.4	14.2	10.3	8.1

TRAM flap: transverse rectus abdominis myocutaneous flap.
